# Leveraging lived experience: a qualitative study on the impact of formerly incarcerated consultants in public policy making to improve safety net services

**DOI:** 10.1186/s12954-026-01484-0

**Published:** 2026-06-06

**Authors:** Jazmin Candelario, Diana Zúñiga, Diamond Lee, Califia Abwoon, Sandy Arevalo, María Casillas Gonzalez, Jason Garcia, Sergio Garcia, Lami Glenn, Gilbert Johnson, Jack Morris, Tue Huynh, Yusef Wiley, Arleen F. Brown, Savanna L. Carson

**Affiliations:** 1 Medical College of Wisconsin, Milwaukee, WI USA; 2 Reentry Health Advisory Collaborative, Los Angeles, CA USA; 3 Tres Lunas Consulting, Long Beach, CA USA; 4 Liberation By Design, Los Angeles, CA USA; 5 Create Realistic Change Inc., Los Angeles, CA USA; 6 Project 180, Los Angeles, CA USA; 7 Pride In Truth, Los Angeles, CA USA; 8 Homeboy Industries, Los Angeles, CA USA; 9 St. John’s Well Child and Family Center, Los Angeles, CA USA; 10 Fear for Breakfast, Los Angeles, CA USA; 11 Timelist Group, Inc., Lancaster, CA USA; 12 Division of General Internal Medicine and Health Services Research, Department of Medicine, David Geffen School of Medicine, University of California, Los Angeles, 1100 Glendon Ave, Suite 1100, Los Angeles, CA 90025 USA

**Keywords:** Criminal law, Incarceration, Socioeconomic factors, Participatory governance, Community reentry, Post-release support, Lived experience, Co-design

## Abstract

**Background:**

Los Angeles County-wide criminal justice reform and policy decision-making focused on reentry and diversion from incarceration usually include only the voices of law enforcement and other public-sector employees, with little input from impacted community members. The Reentry Health Advisory Collaborative (RHAC), founded in 2020 with grant funding, was established to engage formerly incarcerated individuals and their communities in county safety-net health systems. We aimed to assess the impact of RHAC and the importance of formerly incarcerated community input in safety-net programs and policymaking.

**Methods:**

In 2022, following 3 years of RHAC implementation, online qualitative surveys were conducted with RHAC members and reentry service collaborators, including agency staff, nonprofits, and policymakers, to understand RHAC’s influence and how lived experiences inform justice and health systems.

**Results:**

Thirty replies from collaborators (59%) and eight from members (100%) resulted in thematic findings that highlighted benefits of involving formerly incarcerated persons in program and policy decisions—such as their firsthand experiences, focus on root causes, and community and socioeconomic tailoring approaches—and challenges like limited political power, varying receptiveness to ideas and inclusion, and a lack of sustainable funding. Members shared outcomes like motivation, peer support, leadership skills, and advocacy training needs. Recommendations for future inclusion emphasized promoting awareness and strategies for relationships with public-serving institutions, early inclusion for impacting pivotal decision-making, and continued engagement with the community through on-the-ground grassroots efforts. Barriers to reentry included basic needs, access issues, lack of support, and discrimination.

**Conclusions:**

Compensating lived experience in health and justice services promotes inclusive, equitable policies that reflect community needs.

## Introduction

Systemic violence, generational trauma, homelessness, financial insecurity, chronic disease, mental illness, sexual history and identity, and substance use dependencies are examples of issues affecting the incarcerated and reentry populations, highlighting their complex, intersectional health and social needs as well as access disparities [1–3]. As a result, incarcerated and reentry populations face disproportionate morbidity and mortality [3–8]. Racial and ethnic disparities in health care access and health outcomes are also widespread [9–11]. Incarcerated and reentry populations lack access to socially and culturally appropriate social and health services that are coordinated across transitions from incarceration to community release. Additionally, justice-impacted populations face a shortage of educational, training, and career development opportunities, as well as limited employment opportunities. These complex social needs increase reliance on community-based systems of support, or publicly funded services and benefits, including correctional healthcare services affected by high-risk community transitions, whose effectiveness is shaped by cycles of institutional violence and disinvestment in communities of color.

Criminal justice reform—including incarceration diversion, promoting healthy community reentry, and improving health and social services across the justice continuum—is recognized as a priority for equitable justice systems. Including lived experience in participatory governance—a process that empowers stakeholders to directly engage in decision-making, policy development, and resource allocation—is an approach to improving services for those most affected by health or social inequities [12–18]. It harnesses firsthand experience with these systems as expertise to inform service delivery, such as knowledge of service gaps, assets, challenges, and potential solutions. Input from those with lived experience in criminal justice has already been found to advance health outcomes, including through peer-support programs, policy groups, and in research recruitment [14, 18–20], as well as in leadership positions within the public and private sectors [21]. However, the value and incorporation of lived experience within public systems remain unseen, yet are critical for justice-involved populations, as related high-risk transitions are underrealized [16] or ineffective [22, 23]. Public policies often block opportunities for employment and public benefits such as housing and healthcare, and other publicly-funded or lack thereof, for successful reentry [24–26], creating additional and longitudinal public costs and ethical concerns.

Efforts to include individuals with lived experience as advisors on government systems have the potential to improve criminal justice system diversion, reduce incarceration, and enhance community reentry services, thereby improving social and health outcomes for disproportionately impacted populations [13, 21, 23]. Herein, we describe the implementation and qualitative evaluation of a lived experience advisory board in Los Angeles County, the Reentry Health Advisory Collaborative (RHAC), which is made up of 11 formerly incarcerated members who are compensated for providing insights into health policies and programs within public county systems, aiming to bridge the gap between policymaking and the people being served. We explore the roles of this novel collaboration between County health agencies and people with lived experience, including benefits, challenges, and recommendations for health-system community engagement processes to address health disparities, service gaps, and the social needs of reentry populations.

## Setting

Los Angeles County operates one of the world’s largest jails, which provides on-site health and mental health services, with over 15,000 people incarcerated, of whom 40% are awaiting trial and nearly 6,000 are diagnosed with mental health needs [27–29]. One study found that over 60% of Los Angeles County’s jail population with mental health conditions would have been suitable for service-based diversion instead of incarceration [27]. Despite efforts to decrease the jail population and promote diversion, incarceration rates for Black, Latino, and Indigenous communities have stayed disproportionate.

## Development of the Los Angeles Reentry Health Advisory Collaborative (RHAC)

A waiver supported within the Los Angeles County Department of Health Services (LAC-DHS) conducted the section 1115 Medicaid Whole Person Care-Los Angeles (WPC-LA) program from 2016 to 2021, which aimed to integrate social care into healthcare, including building community partnerships for social care pathways [30]. WPC-LA worked to provide coordinated services to vulnerable Medicaid beneficiaries, connecting health, behavioral health, and social services via a community health worker model [31], for high-risk groups facing homelessness, criminal justice involvement, pregnancy barriers, mental health, substance use, and complex conditions [30, 32]. LAC DHS administered WPC-LA, with key stakeholders including LAC departments (Public Health [LAC DPH], Mental Health [LAC DMH], Social Services, Justice), health plans, clinics, and CBOs. A WPC-LA community collaboration team was responsible for community engagement with the high-risk populations to foster effective partnerships between communities, community-based organizations, and healthcare organizations, thereby improving their ability to serve populations with complex health and social needs [33]. This team secured 2020 grant funding to support a lived-experience advisory board, the Reentry Health Advisory Collaborative (RHAC), to engage justice-impacted individuals and their communities. The goal of RHAC was to understand how individuals with criminal justice experience, including those going through high-risk transitions between incarceration and reentry into society, interact with the healthcare system to improve services, reduce recidivism, and cut down on unnecessary healthcare use.

Following grant funding, the WPC-LA collaboration team conducted RHAC member recruitment in 2020 through a public announcement shared with reentry and justice organizations, as well as within community networks facing higher incarceration rates. The recruitment included an RHAC application that asked about past lived experiences, strengths, and interests. The application screening and review process, including an interview with prospective members, included justice-involved members and the WPC-LA engagement team with lived experience [33]. After selection, each member committed to a minimum of 2 years and agreed to fulfill their role on the RHAC by sharing their lived experience with government and non-government partners to inform and participate in policy and program decision-making. RHAC members were provided with formal compensation for their consultation services ($7000 per year for up to 10 h per month) within county services focused on reentry and diversion, as well as for personal and professional development. RHAC selection occurred in Fall 2020, resulting in 11 formerly incarcerated members with knowledge of local justice systems, community re-entry, and related adversity.

RHAC implementation included virtual monthly meetings (due to the COVID-19 pandemic). Initial meetings focused on foundational setting processes, including co-developing a mission statement, values, and vision; the RHAC decision-making process; goals for what to work on; and team relationship-building exercises. The RHAC provided input critical feedback and participation in decision-making spaces that traditionally only include law enforcement and other public sector employees on strategies to address the health and social service needs of the reentry community, including: (1) effective delivery of primary and behavioral health care services in community and correctional settings (2) building capacity of community organizations to increase health and social service continuity for the reentry population (3) the equitable distribution of local funds to culturally competent providers who are serving the reentry population; (4) community asset mapping and a gap analysis of community resources that address the social determinants of health. See Table 1 for examples of RHAC’s invited participation in government-led justice initiatives. Embedding RHAC within government policies and programs resulted from timely symbiotic, parallel justice-related County initiatives, as well as advocacy and connections by the WPC-LA collaboration team. For example, the county had recently completed the Alternatives to Incarceration workgroup to address the impacts of racial justice in the criminal justice system [34–36]. In addition, the 2020 COVID-19 pandemic led to a public health crisis where overpopulation in jails and prisons raised public health concerns in addition to concern over growing awareness of disparities in incarceration. As well, the murder of George Floyd in 2020 sparked public uprisings demanding more accountability to reduce disparities within health systems and justice departments, including taxpayer-related community services and education supporting diversion, and success in reducing unnecessary healthcare costs for reentry populations through proper healthcare and mental health services. This environment fostered funder interest for supporting the work of the RHAC, internal buy-in from Los Angeles County health agency leadership, and advocacy by and connections from the WPC-LA collaboration team to include lived experience in decision-making, which opened the door to its inclusion in these initiatives. Following initial Community Health Care Foundation funding in 2020, RHAC and the collaboration team founders obtained three rounds of Robert Wood Johnson Foundation funding through 2025, totaling nearly $500,000 in RHAC funding.

**Table 1:** Examples of RHAC participation in Los Angeles County government justice initiatives

Initiative	Description	RHAC role/participation
Los Angeles County Alternative to Incarceration Work Group and Office	County initiative addressing the impact of racial justice in the criminal justice system, including service gaps to improve diversion [34]	Development of Alternatives to Incarceration recommendations, workgroup sessions, and community engagement process Formal Consultants, 2025 Alternatives to Incarceration Work Group reconvening
Los Angeles County Justice Care Opportunities Department	The Los Angeles County Care First and Community Investment (CFCI) initiative is a voter-approved measure that allocates funds for alternatives to incarceration and programs. Measure J allocates 10% of the County’s unrestricted funds to address racial injustice through community investments (youth development, job training, small business development, supportive housing services, and alternatives to incarceration) [37]	Voting Member, Los Angeles County Care First Community Investment Advisory Committee Public Comment Participation
Los Angeles County Department of Health Services, Office of Diversion and Reentry	Aims to safely reduce the jail population due to COVID-19 and eventually close Men’s Central Jail by expanding the continuum of care for individuals being diverted/released while ensuring public health and safety. Entitled CEO’s Office COVID-19 Jail Reduction Work Group, also known as the Community Safety Implementation Team	Formal consultants, COVID-19 Jail Reduction Work Group* Formal consultants, Men’s Central Jail Closure Workgroup*
Los Angeles County, Equity, Diversity, Inclusion, and Antiracism (EDIA) Initiative	LAC DHS Office of Diversion and Reentry diverts people with serious mental, physical, and/or behavioral health needs from the Jail and into community-based care. LAC DHS’s department-wide plan is to build a health care system that is fairer and more just for both patients and staff	Strategic Planning Member (Reentry Lived Experience Representative) for EDIA strategic planning process*
Los Angeles County Alternative Crisis Response	Aims to de-escalate behavioral crises and avoid police response, thereby preventing any collateral consequences from a police encounter, arrest, conviction, or incarceration [38] within Los Angeles County Department of Mental Health	Formal consultants, the Alternative Crisis Response Work Group*

*Internal County Meetings (not open to the public)

## Methods

An RHAC-partnered evaluation was conducted to support understanding of the impact of implementing participatory decision-making with formerly incarcerated individuals in Los Angeles County safety-net programs and policymaking. A critical realist [39] and radical research paradigm [40] anchored this study, acknowledging that one’s culture and experiences of structural and systemic barriers can shape a participant’s reality. In addition, community-partnered research methods [41–44] and a logic model for inclusive lived experience [15] supported the development of the design of this work. RHAC members and the research team collaborated on study design, recruitment, interpretation, and dissemination. The [INSTITUTION BLINDED] IRB approved this study (IRB#22-000450).

Two surveys, one for RHAC members and another for RHAC collaborators (including county agencies, non-profits, and other partners who have used RHAC as consultants or had significant interaction with RHAC in various government settings, see Table 1)—were developed through collaboration between a [INSTITUTION BLINDED] research team and with RHAC feedback. These surveys included open-ended questions about common barriers to healthy reentry for justice-involved populations, the value of lived-experience advisory roles within the safety net health system and related government initiatives, as well as demographics and participant characteristics. The questions for both surveys explored RHAC’s role and impact, such as “How has RHAC’s lived experience expertise influenced policy decision-making within city/county justice, health, or social services programs?” Questions also addressed the benefits and challenges of incorporating lived-experience perspectives into government systems and sustainability, asking, “How do you see RHAC playing a long-term role in government systems and policies or programs?” and “What types of policies or decision-making should RHAC be involved in moving forward?” RHAC members were additionally asked a few questions about the personal and professional impacts they experienced since joining.

RHAC member survey eligibility required having served as an RHAC member for more than 1 year and having attended and provided input in both public and internal government meetings. RHAC collaborator survey eligibility included county agency collaborators (e.g., LAC DHS or LAC DPH staff), policymakers (e.g., Board of Supervisors, Public Defender’s office), and community-based organizations serving justice-involved populations that interfaced with RHAC in decision-making. Email recruitment was conducted, and outreach was facilitated by RHAC members or co-founders, who provided contact information for county collaborators, policymakers, and community-based organizations with whom they had interacted in their roles as RHAC members. The survey was distributed between July and September 2022. Both survey types were distributed virtually and completed via Qualtrics or phone interviews. Instead of taking the survey, interviews were conducted virtually with five of the eight RHAC members who were more comfortable sharing their input verbally using the same survey questions, rather than writing. The sessions lasted approximately 1 h. All participants received informed consent and received a $50 gift card.

Qualitative survey results and interview transcriptions were coded using a six-step reflexive thematic analysis for coding and theme development [45–47]. Data familiarization included reading and re-reading the survey results and transcripts. Group discussions between the research team included generating notes on patterns in perspectives and experiences, as well as brainstorming initial coding structures in memos. One primary coder repeatedly read the transcripts, reviewed preliminary content notes, and coded the dataset to delineate common themes. The coder used a data-driven, inductive coding process, focusing on semantic content loosely based on question domains and initial team reflections, and then on latent descriptive coding through clustering, renaming, splitting, or deleting based on similar meanings. Central themes were revised and defined based on testing provisional themes to determine whether patterns were evident across the dataset and how well candidate themes reflected the data. Preliminary themes and results were also presented to the RHAC founders and RHAC members, discussed in four meetings, and refined based on RHAC feedback. The research team reviewed each iteration of the themes, codes, and definitions and recommended revisions or clarification.

## Results

Of 51 RHAC collaborators emailed, 30 completed surveys (59% response rate), and of 8 current RHAC members, 8 completed surveys (100% response rate). Non-responder collaborators included four contacts who had since left their position (1 non-profit, 3 Los Angeles County agency staff), seven community-based organizations, and ten Los Angeles County agency staff. The demographics of the survey respondents are in Tables 2 and 3.

**Table 2:** RHAC County collaborator demographics (*n* = 30)

		*N*	%
Gender	Female	24	80
	Male	3	10
	Other	1	3
	Missing	2	7
Race	Asian American	7	23
	Black/African American	8	27
	White/Caucasian	11	37
	Native American/American Indian/Alaska Native	1	3
	Other	1	3
	Prefer not to answer	2	7
Ethnicity	Hispanic, Latino, or Spanish origin	6	20
Age	18–24	1	3
	25–34	7	23
	35–44	9	30
	45–54	9	30
	55–64	4	13
Justice system involvement (*check all that apply*)	I have been incarcerated in the California Prison System	1	3
	I have been incarcerated in the LA County Jail system	1	3
	I have been incarcerated in another correctional institution	1	3
	I know someone who is or has been incarcerated in the Federal, State, and/or LA County Jail system	25	83
	I have worked within Justice/Correctional systems or have an official role in oversight of these systems	6	20
	Prefer not to answer	4	13
Groups you identify with (*check all that apply*)	Experience with substance use	4	13
	Experience with mental health (living with a diagnosis, etc.)	5	17
	Experience with homelessness	3	10
	Experience with DV/IVP (Domestic, Intimate Partner Violence)	4	13
	Former Life Sentence		0
	Receiver of public benefits/assistance (Cal Fresh, social security, disability, etc.)	2	7
	Chronic disease (diabetes, heart disease, etc.)	4	13
	Disability (physical, cognitive, etc.)	2	7
	LGBTQIA+	4	13
	Veteran	2	7
	Pregnancy and/or parenting	7	23
	Prefer not to answer	14	47
Type of entity you represent	County Health System employee	7	23
	County Justice system employee	3	10
	Social services system, community-based organization, or non-profit employee	11	37
	Other County public services employees (Dept. of Mental Health, Public Health, etc.)	2	7
	Business owner	1	3
	Policymaker, City-level	1	3
	Policymaker, County-level	5	17

**Table 3:** RHAC member demographics (*N* = 8)

		*N*	%
Gender	Female	2	25
	Male	6	75
Race	Asian American	1	13
	Black/African American	3	38
	White/Caucasian	1	13
	Native American/American Indian/Alaska Native	1	13
	Other	3	38
Ethnicity	Hispanic, Latino, or Spanish origin	5	63
Age	25–34	1	13
	35–44	3	38
	45–54	3	38
	55–64	1	13
Justice system involvement (*check all that apply*)	I have been incarcerated in the California Prison System	6	75
	I have been incarcerated in the LA County Jail system	6	75
	I have been incarcerated in another correctional institution	4	50
	I know someone who is or has been incarcerated in the Federal, State, and/or LA County Jail system	4	50
	I have worked within Justice/Correctional systems or have an official role in oversight of these systems	1	13
	Prefer not to answer	1	13
Groups you identify with *(check all that apply)*	Experience with substance use	5	63
	Experience with mental health (living with a diagnosis, etc.)	4	50
	Experience with homelessness	4	50
	Experience with DV/IVP (Domestic, Intimate Partner Violence)	4	50
	Former Life sentence	3	38
	Receiver of public benefits/assistance (Cal Fresh, social security, disability, etc.)	3	38
	Chronic disease (diabetes, heart disease, etc.)		0
	Disability (physical, cognitive, etc.)		0
	LGBTQIA+	1	13
	Veteran		0
	Pregnancy and/or parenting	4	50
	Prefer not to answer	2	25

The results for the overarching categories and themes are presented in Table 4.

**Table 4:** Thematic results

Category	Themes
Benefits of including formerly incarcerated individuals in government policy and program decision-making	Provides first-hand experience in reentry systems and services Continuous focus on the root causes of incarceration in policy and programs (i.e., poverty, education, job opportunities) Promotion of humanistic values in diversion and reentry services Promote community and socioeconomically tailored services
Challenges faced by formerly incarcerated individuals in government policy and program decision-making	Limited political power Variable receptiveness from policymakers and systems for change RHAC sustainability is unknown due to reliance on private funding sources
Common barriers to successful, healthy reentry	Limited availability/accessibility of basic needs for reentry Lack of navigational support for care and resources upon reentry Systematic discrimination assumes a lack of capacity for change in formerly incarcerated individuals
Recommendations for future inclusion of lived experience in policies, services, and programs	Promoting awareness and strategies for relationships with public-serving institutions Early inclusion for impacting pivotal decision-making Continued engagement with the community through on-the-ground grassroots efforts
Professional outcomes of RHAC members related to advocating for criminal justice-impacted individuals*	Motivated by adversity to advocate for criminal justice-impacted individuals Authentic peer support network and resources for the empowerment of formerly incarcerated individuals’ lived experiences Capacity building for leadership skills, interprofessional advocacy training, and knowledge of County systems

*Theme category that was relevant to questions asked to RHAC members only

### Benefits of including formerly incarcerated individuals in government policy and program decision-making

#### Provides first-hand expertise in reentry systems and services

Including RHAC in decision-making provides government decision-makers with direct experience of the justice system and firsthand insights. RHAC members’ lived experiences introduce diverse perspectives to a broad range of government programs and policies related to incarceration, social services, reentry, healthcare and access, substance use disorder treatment, and family welfare issues. Participants noted that members can propose practical policy solutions that affect justice-involved individuals. This helps reduce unintended harm and policy consequences by minimizing biases and strengthening the connection between government officials and the challenges faced by justice-involved communities. Participants also highlighted that this insight helps fill the knowledge gap for policymakers who lack direct experience with criminal justice systems, including the barriers they face, service gaps, and the timing involved in receiving services to meet those needs. As a RHAC collaborator described, “*without the input or voices of people with lived experience, there is a huge gap in knowledge about what actual barriers exist to supporting folks in the system, as well as what truly works for people in terms of achieving well-being and successful outcomes*”. A RHAC member describes their lived experience expertise, “*Experience matters because it gives us insight no textbook can teach. We know where the system fails because we*’*ve been failed by it, but we also know what it takes to get back up and rebuild that knowledge. [It] is rooted in pain, yes, but also in resilience and in the deep wisdom that comes from surviving and thriving. We don*’*t just talk about second chances. We know what it feels like to be denied one*”.

#### Continuous focus on the root causes of incarceration in policy and programs

Participants explained how RHAC focuses on tackling the root causes of justice involvement, such as the criminalization of poverty, health disparities, education issues, and limited career opportunities, by proposing solutions and emphasizing supportive health, community, and social care services that foster justice reform, encourage diversion from incarceration, and support reentry success. As a RHAC member stated, “*This system is inhumane. If a person does not have enough money to pay for shelter, then you know they do not have enough money for food. We punish these people who are desperate. This is why you need people with lived experience. They know how it feels not to have food and shelter*”. Another RHAC member explains how diversion systems are needed: “*Jail hasn’t solved our problem before, and it’s not going to solve it in the future*”. Or, as a RHAC collaborator states, “*The voice of lived experience helps reframe public conversations which were previously centered around the criminal justice system players and responding only to concerns about public safety with additional criminalization to a devastating effect*”.

#### Promotion of humanistic values in diversion and reentry services

Lived experiences of RHAC members help raise awareness of the human impact of disparities in the justice system and reentry, thereby improving understanding and empathy. Members support mediation and cultural brokerage for justice-impacted populations. They also build trust and develop meaningful relationships, which facilitate understanding of the best ways to implement human-centered programs and policies that foster trust with justice-involved populations. As a RHAC collaborator explains, “*RHAC members have been able to humanize the data and policies—to remind decision-makers about the impact their decisions have on human beings, which is incredibly powerfu*l”. Or another collaborator explains the importance, *“Ensuring that people who have historically been excluded from the conversations were sitting at the table and aiding policy creation that creates more empowered and well-resourced communities*”. Another RHAC collaborator described the need for lived experience in other programs, such as homeless services, explaining, “*RHAC should be involved in anything related to anti-homeless policy decisions that give law enforcement ability to arrest, chase out, harass unhoused neighbors or criminalizing survival strategies as the reentry population makes up a significant proportion of our unhoused community members*”.

#### Promote community and socioeconomically tailored services

Participants emphasized that RHAC’s lived experience underscores the importance of developing tailored strategies that effectively address needs, priorities, trust issues, and services connected to structural inequities and social determinants of health. These strategies aim to lower barriers to accessing comprehensive social, health, mental health, and other community services, while also enhancing funding allocation within county program initiatives. As a RHAC member explained their involvement in funding decisions to refocus services on prevention versus criminalization, *“they [were] thinking of committing money to an agency that contracts with police officers. This didn’t match up because they should be contracting out to community members. So, we, [the RHAC] spoke up to remind them of this [and how it went] against any good common sense*”. As a RHAC collaborator also described the community-centered approaches suggested by members to better tailor services to local resources, populations, and environments, explaining, “*They also advocated for a more community-engaged process, including increased participatory budgeting and the use of third-party administrators for County funds that allow expedited and lower barrier solicitations that allow smaller ‘mom and pop’ organizations to apply for County funds*”. Population-specific tailoring also occurred as an RHAC member describes intersectional lived experiences that contributed to their guidance of programs and policies, “*I try my best to use my own lived experience to give advice and voice so the department of corrections can understand and make the system better for those who cannot speak for themselves, including [criminal justice system impacted] gay or lesbian [communities]*”.

### Challenges faced limiting impact by formerly-incarcerated individuals in government policy and program decision-making

#### Limited political power

Participants acknowledged that, since RHAC is a lived-experience advisory committee funded by grants, a major challenge is its lack of decision-making or voting authority in official government roles or appointments. Consequently, RHAC members have limited power to formally apply and leverage their lived-experience expertise. As a RHAC member stated, “*We can advocate, shed light on certain things, hold the county accountable, but if we don’t have a say or vote, where does it really go?*” Or, as a RHAC collaborator states, “*The county has not empowered the RHAC specifically in these settings to create decision-making power or go beyond advisory roles*”. Additionally, the lack of one department or agency that can host RHAC sustainably means that it is “*difficult for RHAC to find a ‘home’ within the county structure*”, as a RHAC collaborator explains. Conversely, some members argued that RHAC was better suited as an independent body because it offered more independence and helped avoid potential conflicts of interest if it was not paid directly by the county, as one RHAC member explains, “*We have no fear of upsetting [people in leadership positions as we are an independent entity and are] able to speak [our] truth to power*”.

#### Variable receptiveness from policymakers and systems for change

Survey respondents described how incorporating lived experiences into systems can lead to frustration due to power dynamics, inaction, perpetuation of the status quo, or, at times, defensiveness toward current institutional structures. These factors were described as promoting intimidation and, at times, triggering re-traumatization for RHAC members attempting to engage in meaningful conversations with policymakers. As a RHAC member stated, “*We get dismissed. What they are proposing as solutions have already failed. I’m beginning to think the county and state are falling into a never-ending cycle*”. As a collaborator explained, “*Challenges include county bureaucracy and gaslighting, which can be super heavy for individuals with lived experiences. Pushing for change in policies and legislation in environments that are oppressive and don’t want to change can be re-traumatizing*”.

#### RHAC’s sustainability is unknown due to reliance on private funding sources

Despite three successful rounds of foundation funding for RHAC’s implementation and maintenance over 5 years, there is a lack of ongoing financial support to enable RHAC to continue its role as a group that supports, advises, and engages in decision-making with government systems on public policy, utilizing lived-experience expertise. A RHAC collaborator explains the limited representativeness of incarcerated persons in decision-making and a desire to be more inclusive in the future: “*I would like to see a large contingent of RHAC members or subcommittees be prioritized in representation (especially) local and county government and public/mental health policy*”. However, sustainable funding for RHAC and the promotion of the benefits of lived experience in decision-making require additional support and systemic change to achieve participatory inclusion. A RHAC collaborator explains, “*It starts with the recognition that this voice is needed at decision-making tables. We are getting there in many spaces, but this is a transformation that will require leadership, as well as an investment of time and effort to implement it well*”.

### Common barriers to successful, healthy reentry for justice-involved populations

#### Limited availability and accessibility of basic needs for reentry

Reentry individuals require urgent attention to their basic needs upon release, including housing, transportation, healthcare and mental health benefits, substance use treatment, career development and employment, and family reunification. Lack of addressing basic needs leads to health impacts, including morbidity and mortality, crime, recidivism, and drug addiction relapse. As one RHAC member stated, “*People can’t make a change unless basic needs are met…food, clothing, and access to healthcare*”, or another RHAC collaborator said, “*Lack of available, affordable, and supportive housing options is a huge barrier*”.

#### Lack of navigational support for care and resources upon reentry

Participants expressed a need for guidance, support, and assistance in navigating comprehensive healthcare, mental health services, and resources to support functional reintegration into society after incarceration and to reduce recidivism, homelessness, and poor health outcomes. Recommendations for navigational support included navigation in obtaining proper identification, such as driver’s licenses and ID cards; completing public benefits applications, including Medicare and Medicaid; navigating the healthcare system, including ensuring medication continuity after release; pursuing professional, educational, and career development; and receiving technology assistance. Navigating these systems can be overwhelming, difficult, or inaccessible, especially when gaps exist in current systems upon release—such as transportation, awareness, and technology literacy. Reentry individuals often face the challenge of "catching up" (RHAC member) after their release. One RHAC member described not being able to know how to use Google Maps upon reentry to get to the DMV, as internet maps were not available before incarceration, saying, “*The system could have done better by providing resources and a network before going out in the free world. You are behind compared to a lot of people. You don’t have the support. It is hard to adjust to the environment*”. Or, as a RHAC collaborator described, “*Simply having folks that walk with them and offer mentorship around things like how to maintain a job*”.

#### Systematic discrimination assumes a lack of capacity for change in formerly incarcerated individuals

Participants explained how policymakers and communities focus on criminalization, creating a gap between those with the power and resources to make changes and those affected by the justice system. This results in structural barriers, outdated policies, and practices that prevent reentry populations from rebuilding new lives after incarceration, which could benefit society. Participants identified racism, discrimination, prejudice, and stigma as reasons for the lack of availability, accessibility, and support for reentry services. As one RHAC member stated, “[*Policymakers assume] that we’ll never change*”, or another RHAC member said, “When I was incarcerated, *there was no reentry support. There was just a stigma, and if you were a felon, because that’s what they called us, felons, convicted felons. We were not allowed to be part of society like everyone else, and we had to fight double even to get just a slightly normal job. We needed to fight a lot harder to get our kids back*”. And as one RHAC collaborator described, “*ongoing stigma post-release*”.

### Recommendations for future inclusion of lived experience in policies, services, and programs

#### Promoting awareness and strategies for relationships with public-serving institutions

To improve the utilization of people with lived experience, participants recommended enhancing awareness of the advisory board’s existence and its potential service, sharing the benefits of inclusion or successes from previous partnerships, and fostering a strategic relationship with state, county, and city department leaders in justice, reentry, health, and social services. For example, as a RHAC collaborator stated, the importance of sharing the benefits of lived experience inclusion, “It starts with the recognition that this voice is needed at decision-making tables. We are getting there in many spaces, but this is a transformation that will require leadership, as well as an investment of time and effort to implement it well”. Another RHAC collaborator mentioned how “policies and decision-making regarding housing, mental health, substance abuse treatment, [and] health and human services” could benefit from lived experience insights.

#### Early inclusion for impacting pivotal decision-making

Participants recognized that early inclusion in programs and policies was essential for integrating lived experience at key pivot points to reduce inefficiency, minimize the risk of ineffective programs and policies with limited reach, acceptability, or appropriateness. As a collaborator explained how RHAC could help better shape community-centered outcomes and services for contracts by being “*provided early drafts of and reviewing all state and federal grants a county agency is going after, which relates to serving marginalized communities… RHAC should not be able to block these grant applications going forward, but rather provide critical insight about how to better structure the asks*”.

#### Continued engagement with the community through on-the-ground grassroots efforts

RHAC members specifically described how lived experience is a skill that needs to be continually honed, emphasizing the importance of ongoing engagement with the community or justice-involved populations to gain up-to-date insights. Participants recommended that RHAC members collaborate with justice-impacted populations and families to understand the latest impacts of public policies and services, thereby informing current needs, priorities, and assets. As an RHAC member stated, “*those most impacted by the issues are best fit to solve them, right? And I would add that while we’re best fit to solve them, we are also often the furthest from the power and resources needed to drive change*”. Or another member said, “*Our lived experience [is important] because we know how damaging the policies [put] in place today [can] destroy the neighborhoods we live in*”.

### Professional outcomes of RHAC members related to advocacy for criminal justice-impacted individuals

#### Motivated by adversity to advocate for criminal justice-impacted individuals

Members are given a platform to share their personal experiences, struggles, and successes, and to advocate for criminal justice policy reform, including promoting diversion, reducing incarceration, and supporting reentry and rehabilitation. As a RHAC member explains, “*I wanted to be a part of the RHAC because of the many injustices, because I wanted to advocate for people like me. I wanted to serve as a voice for those who couldn’t*”. Another RHAC member explains the supportive environment for recognizing the expertise of lived experiences of incarceration and reentry, explaining how healing it could be: “*I remember… learning how my experience is valued… This thing I thought I should be ashamed of is actually valued. Other people don’t know what it feels like to [go to prison.] It’s not what everyone has been through. I learned how to be proud of my experience*”.

#### An authentic peer support network and resources for the empowerment of formerly incarcerated individuals’ lived experiences

A common commitment among formerly incarcerated individuals serving the incarcerated and reentry communities creates a unique space for sharing ideas and resources. This fosters a strong network of people with lived experiences, free from stigma or shame, thereby supporting empowerment. Internal RHAC meetings were supported through structured discussions, facilitation by RHAC founders, and the co-development of rules, mission statements, and values. As an RHAC member states, “*I was able to express myself, not be judged, and to grow with like-minded individuals*”. Another RHAC member explains the emotional impact of building power through collective advocacy of the reentry population, explaining, “*Never have I been in a [space], focused on my experience in and out of these systems and my growth… it meant so much, especially having dealt with shame and choices made in my past*”.

#### Capacity building for leadership skills, interprofessional advocacy training, and knowledge of County systems

RHAC enabled members to engage with policymakers and county directors in spaces where they could speak up and build strategic relationships. A RHAC member explains, “*[The] knowledge [I’ve learned has been invaluable, such as] leadership development, county structures and systems, county budget movement, participatory budgeting, marketing strategies, and more*”. Members expressed a desire to enhance their interprofessional skills through public and advocacy training for use during public meetings. As a RHAC member explains, “*Sometimes it’s hard to articulate your words and with something you are passionate about. Even though I consider myself outspoken, I think some kind of training in public speaking would help me. Being able to articulate yourself gives you a certain degree of credibility and helps you be seen in a more professional light*”.

## Discussion

From this qualitative study, we found that safety-net, criminal justice, and public health system collaborators (both governmental and non-governmental) and RHAC members recognized the values and benefits of including participants with lived experiences of the criminal justice system (such as formerly incarcerated individuals, system-impacted individuals, and their communities) in health and social systems. Their insights bridge the gap between policymaking and the communities they serve, drawing upon their lived experiences with the justice system. RHAC provides specialized expertise, benefits, and value in support of government policies and programs. However, challenges persist in incorporating lived experiences, including securing funding, achieving meaningful inclusion, exercising political influence, and fostering receptiveness to advocacy for systemic change.

Typical government decision-making may not consistently include consultation, support, budgeting, or logistical processes for lived experiences or those most impacted. Some recent notable developments include the state of Washington passed a 2022 bill to incorporate payments for lived expertise in local program and policy design, providing a model for other states [48], or in 2023, the Council of State Governments’ established a Lived Experience Advisory Panel [12] and the 2016 U.S. Department of Housing and Urban Development’s Continuum of Care Programs Interim Rule which requires that a person with lived experience sit on the Program’s Board of Directors [49]. However, RHAC faces challenges in securing power and financial stability beyond private foundation grants for sustainability, and at times has faced limited receptiveness—this can lead to frustration among RHAC members or feelings of tokenization. Tokenization is a concern when incorporating lived experience into policy and program design, where expertise is included but not meaningfully integrated [13, 21]. This frustration can be amplified when there are low expectations or limited belief in the capacity of persons with criminal justice or incarceration histories to change, improve, and grow, even after paying societal debt [21]. RHAC members in our study described the frustrations with limited policy receptiveness, stigma, or lack of awareness for the long-term strategies of diversion and reentry versus short-term strategies and long-term consequences of incarceration. A lack of sustainable funding also hinders long-term goals, strategic planning, and ultimately, the inclusion of policies and programs. Many collaborators recommended further building awareness of RHAC and strategic relationship-building with government and public collaborators to improve the use of RHAC’s expertise and sustainability.

A strength of RHAC, as highlighted by members and institutional collaborators, was its emphasis on promoting positive changes to reduce incarceration by focusing on root causes, humanistic values, the realities of incarceration and reentry, and the needs of diversion. This included storytelling to personalize the success of supportive initiatives in personal development, growth, family, and careers—win–win for both individuals and society—as well as highlighting the barriers to livelihoods that lead to criminalization. As also argued, these accounts of lived experiences allow for “*adapting and revolutionizing service delivery, design and commissioning; and balancing constructed ‘criminal’ narratives*”, as Buck et al. have also described [21]. The personal examples of systematic obstacles, including navigation of community reentry, underscore the importance of exploring multi-pronged health promotion and resources (such as navigation of benefits, health insurance, health education, and literacy promotion), which will be critical to reducing health disparities for not only incarcerated, reentry populations, but populations at-risk of criminalization and in need of diversion programs [3, 8, 24, 50, 51].

RHAC supported personal development, building capacity and purpose in its members, and provided training and support for growth. However, future needs were identified, including capacity building in public speaking and advocacy, and support for similar future programs. The facilitation of the RHAC by its founders was seen as a strength; however, future work may help to understand how best to facilitate and optimize public policy partnerships with lived experience for mutual benefit, understanding, and policy and program development.

This work has some limitations, as it was an exploratory study of experiences involving a lived experience advisory board that served in temporary roles within a single county system. Justice systems and related decision-making can differ significantly across regions, counties, and states, including between private and public health and social systems, so results may vary depending on the setting. Our sample of individuals with lived experience was much smaller than that of the collaborators, due to the size of the RHAC and the larger number of people it engaged with. The methods included open-ended surveys, which led to variations in response depth and breadth, limiting our ability to further explore the responses.

Future research may explore formal frameworks or policy recommendations to sustainably fund and integrate lived experience groups, including cost analyses, such as RHAC. For example, the U.S. Department of Health and Human Services developed a guidebook for “Methods and Emerging Strategies to Engage People With Lived Experience” to improve “federal research, policy, and practice”, including a logic model providing a framework for how lived experience can benefit agencies, programs, and individuals working on programs, as well as individuals with lived experience [15]. Yet, sustainable funding for these methods within government is not recognized and is listed only as a potential opportunity in grant funding.

## Conclusion

Incorporating lived experience into diversion, incarceration, and reentry services helps inform community needs, assets, and preferences, including firsthand experience of incarceration and how to develop services that promote healthful reentry. Lived experience provides a critical focus on the root causes of incarceration, including socioeconomic and environmental factors, and emphasizes human-centered, empathetic approaches to enhance service uptake, accessibility, and effectiveness. Community and social tailoring increases acceptability. Compensating individuals for their lived experience in publicly funded health and justice services supports the comprehensive inclusion of public input from those most affected by health and social disparities. This approach strengthens community-relevant policies and programs, advancing equity within decision-making for publicly funded services used by those most likely to utilize them. Future research could explore formal frameworks or policy recommendations to sustainably invest in and incorporate lived experience, thereby improving policies, services, and practices.

## Data Availability

The datasets used and/or analysed during the current study are available from the corresponding author on reasonable request.

## Abbreviations

RHAC: Reentry Health Advisory Collaborative
LAC DHS: Los Angeles County Department of Health Services
LAC DPH: Los Angeles County Department of Public Health
